# A Cyanotic Newborn with a Pink Right Upper Extremity

**DOI:** 10.1155/2020/8873156

**Published:** 2020-06-29

**Authors:** Matthias Beichl, Andreas Hanslik, Daniel Zimpfer, Judith Rittenschober-Boehm, Katrin Nagl, Ina Michel-Behnke

**Affiliations:** ^1^Division of Pediatric Cardiology, Department of Pediatrics and Adolescent Medicine, Medical University of Vienna, Vienna, Austria; ^2^Division of Cardiac Surgery, Department of Surgery, Medical University of Vienna, Vienna, Austria; ^3^Division of Neonatology, Pediatric Intensive Care and Neuropediatrics, Department of Pediatrics and Adolescent Medicine, Medical University of Vienna, Vienna, Austria; ^4^Division of Pediatric Pulmonology, Allergology and Endocrinology, Department of Pediatrics and Adolescent Medicine, Medical University of Vienna, Vienna, Austria

## Abstract

Aberrant origin of the subclavian artery (SCA) is a well-known vascular anomaly as part of congenital heart diseases with the left subclavian artery (LSCA) being more frequently affected than the right subclavian artery (RSCA). Complete isolation of the SCA is an even more infrequent aortic arch anomaly, occurring in less than 1% for the LSCA and even less for the RSCA. Isolation of the RSCA in patients with d-transposition of the great arteries (D-TGA) is even scanter with only a hand full of cases being reported in the literature. However, isolation of the RSCA has important implications on hemodynamics and surgical strategies. In this case report, we present a newborn patient with D-TGA which presented with distinct differential cyanosis. While the right upper extremity appeared pink with an oxygen saturation of 100%, the rest of the body was cyanotic. At first, this appearance was interpreted as the Harlequin phenomenon during primary care. However, detailed echocardiography revealed an aberrant origin of the RSCA from the right pulmonary artery, which led to the differential cyanosis. The patient underwent arterial switch operation on day of life two including dissection and reimplantation of the RSCA. The special hemodynamic situation of this is discussed in terms of pathophysiology and as well as its impact on perioperative and surgical management.

## 1. Introduction

We present the case of a newborn with d-transposition of the great arteries (D-TGA), where remarkable differential cyanosis initially assumed as the Harlequin phenomenon led to the diagnosis of an isolated right subclavian artery (RSCA). We discuss hemodynamics, embryology, and implications on preoperative and surgical planning.

## 2. Case Presentation

**Figure 1:**
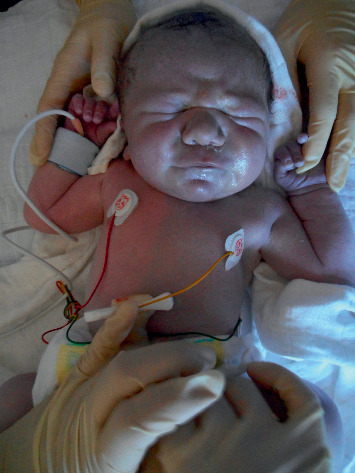
Clinical appearance of the patient during primary care showing marked differential cyanosis.

The patient was a male full-term infant with a birthweight of 3.4 kg and already prenatally suspected complex congenital heart disease (CHD). Fetal echocardiography had suggested D-TGA with a small perimembranous ventricular septum defect (VSD) and possible coarctation of the aorta. The atrial septum defect (ASD) was described as unrestrictive, and no further associated cardiac lesions or vascular abnormalities had been reported. He was delivered by Cesarean section at gestational week 38 and showed sufficient postnatal adaption with an Apgar score of 9, 9, and 9. Throughout primary care, the patient presented with marked differential cyanosis, leaving the right arm and right quadrant of the chest pink with SaO_2_ measured 100% at the right arm, while the rest of the body appeared cyanotic with SaO_2_ levels of 75% (Figure 1). Initially, the differential cyanosis was considered as the Harlequin phenomenon; however, the color change persisted for the next couple of hours precluding the initial clinical diagnosis.

There was no difference in the peripheral pulses with good palpability in all four extremities. Noninvasive blood pressure recordings showed similar systolic blood pressure with values around 80 mmHg, while diastolic blood pressure in the right arm was slightly decreased (35 mmHg) compared to the other extremities (41–44 mmHg).

**Figure 2:**
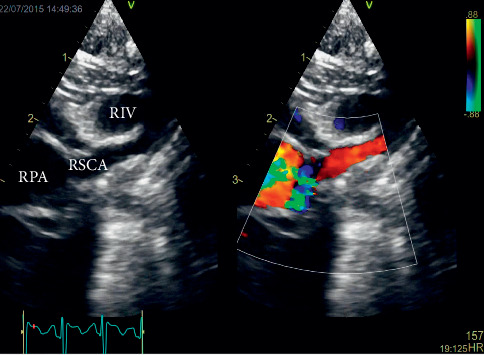
Echocardiography of the newborn with B-image on the left and a simultaneous color Doppler on the right; in a high right parasternal long axis, an aberrant origin of the right subclavian artery (RSCA) from the right pulmonary artery (RPA) could be displayed with course towards the right arm. RIV, right innominate artery.

**Figure 3:**
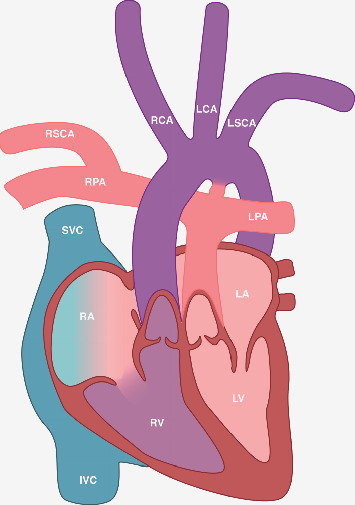
Schematic illustration of a d-transposition of the great arteries (D-TGA) in combination with an aberrant origin of the right subclavian artery (RSCA) from the right pulmonary artery (RPA). IVC, inferior vena cava; LA, left atrium; LCA, left carotid artery; LPA, left pulmonary artery; LSCA, left subclavian artery; LV, left ventricle; RA, right atrium; RCA, right carotid artery; RV, right ventricle; SVC, superior vena cava.

Transthoracic echocardiography revealed a parallel course of the great arteries. The artery originating from the left ventricle bifurcated into the right and left pulmonary arteries, whereas the artery originating from the right ventricle was determined as the aorta with a left-sided aortic arch. These findings were consistent with D-TGA with anterior-posterior alignment of the great arteries. A small ASD with a size of 4 mm as well as a ductus arteriosus (DA) with predominantly left-to-right shunting, but some right-to-left shunt during systole, was displayed. Detailed echocardiographic evaluation revealed a left-sided aortic arch with three head/neck vessels. Both coarctation of the aorta as well as a perimembranous VSD, which had been described by fetal echocardiography, could be ruled out during the examination. Notably, neither a branching of the first vessel nor an origin of RSCA distal to the left subclavian artery (LSCA) in terms of a lusoria artery could be visualized. However, an anomalous artery could be displayed in multiple views originating from the right pulmonary artery (RPA) with course between the superior vena cava and aorta towards the right arm (Figure 2). The final diagnosis was D-TGA with aberrant origin of the RSCA from the RPA (Figure 3).

PgE1-infusion (alprostadil) was started shortly after delivery with 15 ng/kg/min. The initial distinct cyanotic appearance, together with a peripheral saturation of 75% and the existence of a small ASD, led to the consideration of a balloon atrial septostomy (BAS). However, as the left-to-right shunt through the ASD could clearly be demonstrated to be unrestricted allowing adequate mixing, as blood gas analysis did not show metabolic acidosis, and as cerebral oxygen saturation remained stable, BAS was not performed, eventually. These findings were consistent with an increase of the peripheral saturation up to 83% with advancing postnatal cardiorespiratory adaption.

On day 2, the child underwent arterial switch operation. Intraoperatively, the surgeon could visualize the RSCA originating from the RPA at the level of the distal main right branch. Ligation and dissection of the RSCA was performed with subsequent reimplantation to the ascending aorta.

Postoperatively, blood pressure differences have not been observed and neither paleness nor temperature differences were noted. One year after surgery, the patient is presenting in excellent clinical condition with appropriate growth of the formerly affected extremity and without any differences in peripheral pulses or blood pressure.

Written informed consent was obtained from the patient's parents for publication of the case report and the newborn's image.

## 3. Discussion

Aberrant origin of the subclavian artery (SCA) is well known to be part of CHD, especially when the aortic arch demonstrates hypoplasia or obstruction. Diagnosis is made mostly by echocardiography prior to surgery and eventually confirmed by CT- or MRI scans. Anomalous origin and course are more common for the LSCA than for the RSCA. While in the majority of anomalies of the SCA blood supply is provided from the aorta, origin of either the RSCA or LSCA from the pulmonary artery (PA) is extremely rare.

In the presented case, unusual differential cyanosis led to the diagnosis of an isolated RSCA in a newborn with D-TGA. The combination of a D-TGA with an isolated RSCA has only been reported in a few cases [1, 2]. However, this vascular anomaly can have significant influence on the management and therapy of these children.

Isolation of the RSCA can be explained on the basis of Edwards' hypothetical double arch [3]. Within the normal embryological process, the RSCA develops from connection of the right 7^th^ intersegmental artery with the right 4^th^ aortic arch. The right 6^th^ aortic arch normally forms the RPA and the right DA, from which the latter usually involutes. In the presented case involution of the right 4^th^ aortic arch resulted in migration and connection of the right 7^th^ intersegmental artery to the right 6^th^ aortic arch, which further led to an aberrant origin of the RSCA from the RPA via a right persisting DA.

Hemodynamic consequences of this rare finding differ widely, depending on the filling characteristics. While in otherwise normal biventricular circulation with normal pulmonary and systemic resistance, blood flow is directed towards the PA and will induce the clinical findings of diminished pulses and lower blood pressure at the right arm. In the scenario where the DA remains open, the RSCA presents as a systemic-to-pulmonary shunt accepting blood from the vertebrobasilar system and donating to the PA (the pulmonary steal phenomenon), leading to pulmonary overcirculation with risk of congestive heart failure.

As in our patient, a neonate with D-TGA, pulmonary pressure and resistance are high and direction of blood flow could be demonstrated as reversed flow from the RPA to the RSCA. Keeping in mind that the PA in D-TGA is connected to the left ventricle with fully oxygenated blood, saturations were measured 100% and pressure differences to the systemic circulation did not occur. Both carotid arteries as well as the LSCA fill from the aorta, which supplies the systemic circulation with deoxygenated blood and thereby gives cyanotic coloring of the skin perfused by these vessels.

Initially, the distinctly delimited differential cyanosis led to the assumption of the Harlequin phenomenon, which is a sudden change in skin color in newborns due to sympathetic autonomic dysregulation of the peripheral vascular tonus [4]. However, the Harlequin phenomenon often shows gravity dependency of the color change and usually resolves very quickly, which was not the case in the presented patient, and therefore, isolation of the RSCA was suspected.

Differential cyanosis, as described in our patient, can more frequently be encountered in a totally different hemodynamic situation, namely, in newborns with pulmonary hypertension (PH) and persistent ductus arteriosus (PDA), interrupted aortic arch, or coarctation of the aorta. These situations have, in common, that desaturated blood flows into the descending aorta via a right-to-left shunt over the PDA, which may cause cyanosis of the lower body half. In case of a postductal origin of the LSCA, this can lead to a similar differential cyanosis as in the presented case. However, since the diagnosis of D-TGA had already been described prenatally in this patient, PH with a PDA could be ruled out in this case, as this would have led to a reverse differential cyanosis. Reverse differential cyanosis can occur in infants with D-TGA with PH or coarctation of the aorta, which causes increased right-to-left ductal shunting of saturated blood and thereby higher postductal peripheral saturation. PH in patients with D-TGA is a critical hemodynamic condition, as increased vascular resistance leads to a predominant right-to-left shunt across the DA, which causes a decrease in pulmonary blood flow and, therefore, an increase of the cyanosis [5]. In case of a postductal origin of the LSCA, this hemodynamic situation can cause a reverse differential cyanosis of the right upper extremity, as oxygenated blood is directed across the DA via a right-to-left shunt, while the right arm is supplied with desaturated blood.

The amount of differential cyanosis between the different skin areas depends on mixing of blood at the atrial and ductal level. Our patient had only a small ASD, which allowed systemic saturations of 75% to 83%. This could be clearly distinguished from the fully oxygenated RSCA, supplying the right arm and right chest wall.

The accurate diagnosis has several implications in the medical care of these patients.

It is very important that invasive blood pressure monitoring on the neonatal intensive care unit and during heart surgery should not be performed on the right arm, as these pressures reflect the hemodynamics in the pulmonary rather than the systemic circulation. Furthermore, measurement of invasive blood pressure on the right arm would lead to discontinuous monitoring during ligation and reimplantation of the RSCA.

For transcutaneous SaO_2_-monitoring, cerebral oxygenation is the most critical parameter and would, in the setting of D-TGA with RSCA, be reflected most accurately by recording the left arm tracing. It is also this saturation that gives most information on whether a balloon atrioseptostomy should be performed for better mixing between the circulations.

The surgical decision to reconnect the RSCA to the aortic arch, as done in our case, may be preventive for subclavian steal syndrome on the long run, as described in one case with D-TGA and isolated RSCA [2] or more frequently in other CHD with isolated LSCA [6]. Equally leaving the RSCA would account for the pulmonary steal phenomenon as soon as the pulmonary pressure and resistance normalize after arterial switch operation.

Clinical appearance of differential cyanosis presenting as the Harlequin phenomenon should be taken seriously in neonates with cyanotic CHD and not brushed aside. Meticulous echocardiographic evaluation and eventually additional CT or MRI investigation provides the diagnosis of anomalies of the head neck vessels that may influence hemodynamics, perioperative preparation, and surgical strategy significantly. Pathophysiology of aberrant or isolated RSCA affords cardiovascular thinking also in nonspecialized neonatology units.
